# Analytical study of the psychosocial impact of malocclusion and maxillofacial deformity in patients undergoing orthodontic treatment

**DOI:** 10.25122/jml-2020-0022

**Published:** 2021

**Authors:** Anurag Rai, Minti Kumari, Tanoj Kumar, Shweta Rai, Himali Gupta, Renu Singh

**Affiliations:** 1. Department of Orthodontics, Patna Dental College and Hospital, Bankipore, Patna, India; 2. Department of Public Health Dentistry, Patna Dental College and Hospital, Patna, Bihar, India; 3. Department of Oral Pathology and Microbiology, Patna Dental College and Hospital, Patna, Bihar, India; 4. Department of Oral and Maxillofacial Surgery, Buddha Institute of Dental Sciences and Hospital, Kankarbagh, Patna, Bihar, India; 5. Department of Orthodontics, KD Dental College and Hospital, Mathura, Uttar Pradesh, India

**Keywords:** craniofacial anomalies, dental aesthetics, facial aesthetics

## Abstract

Patients whose with facial appearance involves dental anomalies and malocclusion face an increased prevalence of various psychosocial problems such as a high level of social anxiety, social avoidance, and low quality of life. This study investigates the patients with craniofacial anomalies and their psychological adjustment concerning the facial and dental appearance. It also evaluates the expectations of this patient group from the orthodontic treatment. Two steps were done in this study. In the first step, translation and validation of the Derriford Appearance Scale (DAS59), The Psychosocial Impact of Dental Aesthetics Questionnaire (PIDAQ), and Patient Expectation from the Orthodontic Treatment (PEOTQ) questionnaires into Maithili were done, and then the main study was conducted using these valid questionnaires. This was a cross-sectional study conducted on the patients with congenital craniofacial anomalies visiting the orthodontics department of Patna Dental College and Hospital, Patna (Bihar). All the patients received the Maithili DAS, Maithili PIDAQ and Patients' Expectation from the orthodontic treatment questionnaires. The Maithili version of DAS59, PIDAQ and PEOTQ were developed with outstanding reliability and validity. A significant difference between PIDAQ (p<0.001) and DAS59 scores (p<0.001) was found. In females, the total PIDAQ score was significantly higher as compared to males, but there was no association of DAS scores with gender. Place of residence showed no association with PIDAQ and DAS59 scores in patients. Patients and controls had significant differences between various items, and a comparison was made in terms of expectation from the orthodontic treatment. Altered facial and dental appearance in patients with craniofacial anomalies showed a significant psychological impact.

## Introduction

A craniofacial malformation results in a serious impairment of the normal anatomy of the skull, jaws, and the adjacent soft tissues and is an anomaly of embryonic development [1]. These are the anomalies of head and face that interfere with the physical and mental wellbeing of the individual [2].

The craniofacial structures are largely derived from the neural crest cells, a transient group of multipotent cells specified along the dorsal aspect of the neural tube, delaminated from the neural tube via an epithelial-mesenchymal transition [3]. These cells migrate in streams along specific body segments and subsequently differentiate under the guide of many signaling pathways throughout their journey. The cranial neural crest cells delaminate from the anterior segment of the folded neural tube and migrate in a single wave to give rise to the majority of the craniofacial structures. The migration of cranial neural crest cells is influenced by their physical contact with one another [3].

Alteration in any of these processes serves as a basis for these anomalies, collectively termed as neurocristopathies. The craniofacial structures are associated with an inconsistent number of birth defects due to the niceties involved in the genesis of a diverse collection of tissues present in a relatively small volume [4].

The gamut of craniofacial anomalies is diverse, and the most common conditions include cleft lip and/or palate, Crouzon’s syndrome or Apert’s syndrome (craniosynostosis), Treacher Collins syndrome (otomandibular anomalies), holoprosencephaly, Stickler syndrome, and fetal alcohol syndrome [5]. The clinical features include a spectrum of deformities of the craniofacial region, including cranium and cranial sutures, deformity of skull shape and facial bones including the maxilla, mandible, zygomatic arches, nose, eyes, ears, lips, and teeth [4–10].

The etiology of craniofacial anomalies is not entirely understood, but it is said that it is multifactorial, which includes both genetic and environmental factors. Genetic factors include mutations and polymorphisms or chromosomal aberrations like Down’s syndrome [6–8]. Environmental factors consisted of nutritional factors (e.g., vitamins, teratogens), medications (aspirin and valproic acid), viral infections like rubella, maternal smoking, and alcohol consumption [8, 9].

Craniofacial anomalies, along with systemic defects, also lead to facial deformities, including deformity of skull shape, facial bones including maxilla, mandible and zygomatic arches, nose, eyes, ears, lips and teeth [10–12]. The patients with abnormal facial appearance often have to face discrimination in society as they are considered to be less attractive and are often considered as less capable, less intelligent and less honest, leading to various psychosocial problems such as a high level of social anxiety, social avoidance and affect the quality of life [13–15]. The appearance problem in this patient group is further compounded by an increased prevalence of dental anomalies and malocclusion [16–18].

The objectives of this study were to assess the psychological adjustment in relation to facial appearance, the psychological impact of dental appearance in patients with craniofacial anomalies, and the expectations of this patient group from the orthodontic treatment.

## Material and Methods

This was a cross-sectional comparative study conducted at the Department of Orthodontics, Patna Dental College, Patna (Bihar), from January 2019 to November 2019. The study was conducted in two steps. In the first step, we included the translation and validation of the instruments – Derriford Appearance Scale (DAS59), The Psychosocial Impact of Dental Aesthetics Questionnaire (PIDAQ), and Patient Expectation from the Orthodontic Treatment (PEOTQ) questionnaires in the target population. In the second step, we included the patients with craniofacial anomalies who received orthodontic treatment using these validated instruments.

For the first step, we included three instruments – DAS59, PIDAQ and PEOTQ. A pack of questionnaires that included all these three instruments was developed. The content of these three packs was as follows:

**Pack 1: (DAS59)**

1. **Derriford Appearance Scale (DAS59)** – consisting of 59 items, each item response is marked based on a Likert scale from 1 to 4, with 1 indicating “almost never” and 4 – “almost always”.
2. **General Health Questionnaire (GHQ12)** – consisting of 12 items. Each item response is marked based on a Likert scale from 1 to 4, with 1 indicating “not at all” and 4 “much more than usual”. Beck “s Anxiety Inventory (BAI) and Beck’s Depression Inventory (BDI) consist of 21 items each. In BAI, each item response is marked based on a Likert scale from 1 to 4, with 1 indicating “not at all” and 4 “severely, I could barely stand it”. In BDI, each item response is marked based on a four-point Likert scale.

**Pack 2**

1. PIDAQ – consisting of 23 items arranged in four domains. Each item response is marked based on a Likert scale from 0 to 4, with 0 indicating “not at all” and 4 “very strongly”.
2. Index of Orthodontic Treatment Needs Aesthetic Component (IOTN-AC) – Index of Orthodontic Treatment Needs Aesthetic Component (IOTN-AC) consisting of 10 standard malocclusion photographs arranged according to severity. The respondent was asked to tick the photograph that resembles his/her teeth arrangement [16].
3. The perception of occlusion scale (POS) – consisting of six items. Each item response is marked based on a Likert scale from 0 to 4, with 0 indicating “not at all” and 4 indicates “very strongly” [17].

**Pack 3: Expectation from the orthodontic treatment**

The questionnaire included demographic information followed by the items. The participants were asked to mark on a line that has ten equal intervals. This final version was back-translated by two independent translators in the original language who had sufficient knowledge of the target language. As in the previous step, this back translation was synthesized into a final version by a third independent translator with similar skills.

An expert committee comprising translators, orthodontists and representatives of the target population was formulated, and this back-translated final version was presented to them. Then, this questionnaire was tested on 50 participants, and they were asked to rephrase each item using their own words to identify that each item can be understood. A final adjustment was made, and by consensus, the final version was developed.

One hundred twelve patients who satisfied the selection criteria were included in the study. Out of 112, only 102 agreed to participate in the study. Adult patients with craniofacial anomalies of the age group 18–30 years and patients who presented for the first time for orthodontic treatment were included in the study. Patients that were mentally retarded, blind, with acquired or traumatic facial disfigurement, and patients aged less than 18 years or more than 30 years were excluded from the study.

A reliability retest was also conducted. Fifty participants out of the total were randomly selected using a table of random numbers after an interval of 4 weeks.

The data were entered into the SPSS software, version 19. The reliability of the scale was tested by Cronbach’s alpha coefficient and coefficient correlation. The retest reliability was also tested using Spearman’s correlation coefficient between items and the total score of the scale.

## Results

A total of 252 individuals with an equal number of males and females were included. The age group ranged from 18 to 29 years with an average of 22.33±2.114. The response rate was 100% with all valid, fully completed questionnaires.

### Results for translation and validation of PIDAQ

Out of the total, 123 patients demanded orthodontic treatment. Table 1 shows the association between IOTN-AC scores and sub-domains of the Maithili version of PIDAQ using the Kruskal–Wallis test, showing the mean ranks for each score range of IOTN-AC and total statistical analysis.

**Table 1.** Association between IOTN-AC scores and sub-do­mains.

IOTN-AC	**Social Impact**	**Dental Self Confidence**	**Psychological Impact**	**Aesthetic Impact**	**Dental Self-consciousness**
1	82.10	93.50	73.50	92.20	82.88
2	1170.30	127.31	133.55	104.54	126.70
3	173.4	140.25	168.36	162.73	153.62
**4>4**	173.85	166.4	180.46	174.20	175.88

Table 2 indicates the association between POS scores and sub-domains of the Maithili version of PIDAQ using the Kruskal–Wallis test, showing the mean ranks for each score range of POS and total statistical analysis.

**Table 2.** Association between POS scores and sub-domains.

**POS Scores**	**Social Impact**	**Dental Self Confidence**	**Psychological Impact**	**Aesthetic Impact**	**Dental Self-consciousness**
**0-1**	78.88	76.21	67.62	87.56	86.0
**9 Feb**	109.89	122.01	116.57	97.56	107.99
**10 may**	135.98	144.05	127.02	148.00	125.45
**9>9**	189	175.45	201.98	185.12	192.98
**Chi-Square Value**	77.80*	67.31 *	118.35*	76.52 *	79.90 *
* Signifies Asymp Sig 0.000					

Table 3 shows the differences between subjects who are willing and those who are not willing to undergo an orthodontic treatment in relation to the sub-domains of the Maithili version of PIDAQ.

**Table 3.** Differences between subjects who are willing and those who are not willing to undergo an orthodontics treatment.

Maithili PIDAQ	**Willing**	**Non-willing**	**Mann-Whitney U**
**Social impact**	167.21	88.32	2997.0*
**Dental self Confidence**	156.98	96.65	408.00*
**Psychological Impact**	187.14	66.96	324.00*
**Aesthetic Impact**	154.89	99.03	4405.90*
**Dental Concern**	159.87	94.16	3764.00*

### Results for translation and validation of DAS59

The clinical sample consisted of 111 females and 101 males. The age group ranged from 18 to 29 years with an average of 23.08±1.69. The non-clinical sample consisted of 112 males and 100 females with an age group from 18 to 29 (mean age of 23.13±2). The response rate was 100% with valid, fully completed questionnaires. Retest questionnaires also had a 100% response rate.

Tables 4 (a and b) show the difference between domains, the main scale and other scales in patients who are not concerned about their appearance and those with appearance problems by using Mann-Whitney U Test and showing the correlation between DAS59, BDI, BAI and GHQ12. Demographic data and craniofacial anomalies of patients are shown in Tables 5 and 6, respectively.

**Table 4 (a).** Mann-Whitney U test for all scales.

**Scale as domain**	**Mann-Whitney U Test**	**Asymp Sig**
**Das 59**	566.00	0.000 *
**GSC**	956.500	0.000 *
**SSC**	551.01	0.000 *
**SBSC**	1394.00	0.000 *
**NSC**	984.500	0.000 *
**FSC**	613.500	0.000 *
**PHY**	2817.500	0.000 *
**OTH**	2954.00	0.000 *
**BDI**	481.00	0.000 *
**BAI**	783.500	0.000 *
**GHQ**	1292.00	0.000 *

**Table 4 (b).** Significance of the correlation coefficient.

			**Total**	**BDI**	**BAI**	**GHQ**
**Spearman’s rho**	**Das 59**	**Correlation Coefficient**	1.000	0.759	0.727	0.736 **
		**Sig**	-	0.000	0.000	0.000
	**BDI**	**Correlation Coefficient**	0.758	1.00	0.764	0.659 **
		**Sig**	0.000	0.000	0.000	0.000
	**GHQ**	**Correlation Coefficient**	0.752	0.695	0.689	1.000
		**Sig**	0.000	0.000	0.000	0.000

* Correlation is significant at a p-value of 0.001.

**Table 5.** Demographic data.

**Subjects**	**Sex**	**Age**	**Educational Level**	**Rural/urban**
**Patients (102)**	M=53, F=49	24.68±2.46	15.08±1.82	R=48, U=54
**Controls (102)**	M=52, F=50	24.89±2.65	15.35±1.74	R=47,U=55

**Table 6.** Abscence of follow-up patients who participated in the study according to the diagnosis of craniofacial anomaly.

**Sno.**	**Craniofacial anomaly**	**N**
**1**	Isolated Cleft lip/palate	46
**2**	Isolated Craniosynostosis	23
**3**	Hemi facial microsomia	12
**4**	Ectodermal Dysplasia	2
**5**	Cleidocranial Dysplasia	2
**6**	Treacher’s Collins Syndrome	7
**7**	Pierre robin Syndrome	5
**8**	Crouzon’s Syndrome	3
**9**	Apert’s Syndrome	2

### Results for the main study on adult patients with congenital craniofacial anomalies 

When a comparison between the scores of DAS and PIDAQ questionnaires was made among patients and controls, there was a significant difference between PIDAQ scores – 33.25±9.45 for patients and 27.52±5.67 for controls, p<0.001) and DAS59 scores (mean score for patients was 159.16±31.54 and 77.64±6.57 for control, p<0.001) (Table 7).

**Table 7.** Differences between the DAS59 and PIDAQ scores in patients and controls.

Items	**Group**	**Mean**	**Sig (2-tailed)**
**GSC**	1	47.28±10.6	<0.001
	0	20.89±3.4	<0.001
**SSC**	1	58.22±14.6	<0.001
	0	23.7±2.30	<0.001
**SBSC**	1	24.27±5.96	<0.001
	0	8.76±2.07	<0.001
**NSC**	1	9.63±3.14	<0.001
	0	16.89±1.15	<0.001
**FSC**	1	12.02±2.50	<0.001
	0	4.65±1.05	<0.001
**PHY**	1	5.71±1.56	<0.001
	0	2.56±0.97	<0.001
**Total score**	1	157.13±31.45	<0.001
	0	77.45±6.45	<0.001
**PD1**	1	12.83±3.10	<0.001
	0	11.12±2.91	<0.001
**PD2**	1	7.1±3.26	<0.001
	0	6.12±1.74	<0.001
**PD3**	1	6.23±2.68	<0.001
	0	5.04±1.89	<0.001
**PD4**	1	6.86±2.75	<0.001
	0	5.26±1.65	<0.001
**PD5**	1	8.54±4.02	<0.001
	0	6.64±1.88	<0.001
**Total Score**	1	41.56±9.54	<0.001
	0	34.18±5.65	<0.001

The educational level was not associated with DAS59 except for the PHY sub-domain, which was related positively to the educational level. The effect of gender on PIDAQ and DAS59 scores in controls was described in Table 8b. Patients with a high educational level had high PHY scores (Table 9), and it was negatively related to PD2, PD4, PD5, and total PIDAQ scores. The higher the educational level of the patient, the lower the scores of the PD2, PD4, PD5 sub-domains and total PIDAQ score (Table 10). When considering objectively and subjectively the severity of the condition, there was no statistically significant difference in self-assessment of the severity of his/her condition when compared to assessment by a clinician (Table 11).

**Table 8 (a).** Effect of gender on PIDAQ and DAS59 scores in patients.

**Domain**	**Sex**	**Mean**	**Std Deviation**	**Std Error Mean**	**Sig**
**Gsc**	M	49.26	10.782	1.4	0.997
	F	49.02	10.896	1.545	0.997
**SSC**	M	58.77	14.086	1.89	0.721
	F	57.65	14.257	2.00	0.721
**SBSC**	M	24.34	5.928	0.981	0.759
	F	24.06	6.024	0.785	0.758
**NSC**	M	9.54	3.045	0.473	0.69
	F	9.77	3.128	0.472	0.69
**FSC**	M	12.32	3.15	0.384	0.389
	F	11.09	2.505	0.563	0.389
**PHY**	M	5.88	1.684	0.225	0.371
	F	5.78	1.59	0.232	0.371
**TOTAL**	M	193.77	30.574	4.83	0.78
	F	151.59	30.896	4.598	0.78
**PD1**	M	12.56	2.75	1.01	0.621
	F	13.08	3.256	0.453	0.620
**PD2**	M	6.78	3.014	0.486	0.524
	F	7.24	3.690	0.543	0.523
**PD3**	M	5.56	2.25	0.385	0.001
	F	7.06	3.89	0.46	0.001
**PD4**	M	6.43	2.653	0.326	0.005
	F	7.67	2.768	0.452	0.005
**PD5**	M	8.97	4.125	0.572	0.352
	F	8.68	4.206	0.596	0.352
**Total**	M	40.3	7.958	1.103	0.025
	F	43.73	10.546	1.548	0.026

**Table 8 (b).** Effect of gender on PIDAQ and DAS59 scores in controls.

**Domain**	**Sex**	**Mean**	**SD**	**SEM**	**Sig**
**GSC**	M	21.76	3.124	0.382	0.991
	F	21.83	3.326	0.514	0.992
**SSC**	M	22.5	2.578	0.416	0.68
	F	22.6	2.146	0.332	0.671
**SBSC**	M	8.78	2.324	0.329	0.485
	F	8.57	1.987	0.261	0.496
**NSC**	M	16.95	1.31	0.243	0.576
	F	16.86	1.27	0.237	0.558
**FSC**	M	4.54	0.823	0.092	0.368
	F	4.87	1.342	0.230	0.368
**PHY**	M	2.69	0.796	0.132	0.508
	F	2.74	1.105	0.216	0.504
**Total**	M	77.22	6.794	0.998	0.941
	F	77.47	6.418	0.979	0.941
**PD1**	M	11.21	2.766	0.397	0.814
	F	11.15	3.008	0.369	0.816
**PD2**	M	6.03	1.683	0.186	0.575
	F	6.24	1.891	0.195	0.575
**PD3**	M	5.21	1.876	0.336	0.326
	F	4.76	1.873	0.327	0.328
**PD4**	M	5.45	1.479	0.243	0.21
	F	5.06	1.696	0.224	0.211
**PD5**	M	6.92	2.227	0.238	0.32
	F	6.52	1.731	0.291	0.328
**Total**	M	35.02	5.281	0.805	0.506
	F	33.73	6.013	0.794	0.505

**Table 9.** Effect of age and educational level on DAS59 scores in patients.

**E Level**	**Test**	**PD1**	**PD2**	**PD3**	**PD4**	**PD5**	**Total score**
**Age**	**Pearson correlation**	-0.213	-0.281 **	-0.106	-0.369 **	-0.352 **	-0.294 **
	**Sig (2 tailed)**	0.164	0.002	0.314	0	0.005	0.002
	**Pearson correlation**	-0.124	0.103	-0.001	-0.141	-0.02	-0.036
	**Sig (2 tailed)**	0.574	0.593	0.992	0.431	0.784	0.673

**Table 10.** Effect of age and educational level on PIDAQ scores in patients.

E Level	**Test**	**PD1**	**PD2**	**PD3**	**PD4**	**PD5**	**Total score**
**Age**	**Pearson Correlation**	-0.231	-0.315 **	-0.146	-0.362 **	-0.274	-0.313
	**Sig (2-tailed)**	0.05	0.002	0.331	0	0.006	0.002
	**Pearson Correlation**	-0.121	0.142	-0.001	-0.078	-0.02	-0.041
	**Sig (2-tailed)**	0.254	0613	0.978	0.431	0.842	0.672

**Table 11.** Comparison of the objective and subjective assessment of severity among patients.

**Parameter**	**Mean**	**Std. Devi**	**Std. Error Mean**	**95% CI**		**Sig-**
				**Lower**	**Upper**	
**Pair 1 OS1-SS1**	-0.03	0.978	0.096	-0.18	0.16	0.691
**Pair 2 OS2-SS2**	-0.20	0.942	0.092	-0.43	0.01	0.043

The objective and subjective severity were significantly associated (positively) with DAS59 scores and all sub-domains except NSC, which had a negative association. With the increase in objective and subjective assessment of severity, the DAS scores also showed an increase, except for NSC.

Studying the impact of subjective and objective severity on PIDAQ scores, it was noted that the subjective severity was significantly associated with the PD3 sub-domain. With a higher subjective assessment of severity, the PD3 sub-domain scores increased, whereas only the second item of objective severity was significantly associated with the PD3 sub-domain.

## Discussion

When applying to different regional, ethnic and social settings, it is of utmost importance that the questionnaire measures the same construct with the same accuracy. Various studies highlight the common pitfalls and suggest recommendations [20, 21]. In this study, a recent rigorous protocol by Gjersing *et al.* for cross-cultural adaptation of research instruments was used [22]. This protocol involved language, setting, time and statistical considerations.

In the validation of the Psychosocial Impact of Dental Aesthetic Questionnaire, our translation incorporated five domains compared to the original scale and Chinese translation, where 4 domains were present. When subjected to the component factor analysis of the principal component with orthogonal rotation, 5 factors were extracted. Further, the screen-plot confirmed the extraction of 5 components, and they could explain 73.26% of the total variation.

Self-consciousness is greatly related to a preoccupation with oneself and a feeling that oneself is observed by others [23, 24]. All the items that fall in the fifth component were coincident with this idea. Therefore, the “dental self-consciousness” nomenclature was used. One item “I sometimes catch myself holding my hands over my mouth to hide my teeth” has good factor loading under psychological impact as compared to the social impact. This may be due to different perceptions and understanding of these concepts by Indian young adults as compared to other population groups. Similarly, one item from the “dental self-confidence” domain of the original instrument “I like to show my teeth when I smile” had good factor loadings in the “aesthetic concern” domain, again attributed to conceptual differences among various populations. The scale had good criterion validity as its domains correlated well with the IOTNAC and POS scales, the individuals having high PIDAQ values, directly proportional to the IOTN-AC and POS values (Tables 1 and 2). The scale showed excellent responsiveness as there were significant differences between the scores of individuals who had demanded and who did not demand orthodontic treatment in all sub-domains (Table 3).

The construct validity of the Methili DAS59 questionnaire was studied under convergent and discriminant validity. Highly significant differences between the clinical population having appearance concerns and nonclinical population with no facial concerns were demonstrated by discriminant validity. Convergent validity was confirmed by a significant correlation between DAS59, BDI, BAI and GHQ (Table 9).

The instrument has a good validity as described in the original version due to the use of an open-ended questionnaire, which was used to develop a close-ended questionnaire. Face validity was found to be satisfactory by virtue of relevant questions and subjective assessment [6]. To establish construct validity, 100 participants were asked to fill in the questionnaire followed by an interview with a researcher on the theme of the questionnaire, which was recorded and filled in by a second researcher. This was done to assess whether the subject is expressing what he/she knows correctly about the theme and whether the instrument is capable of measuring the underlying construct. There was an excellent agreement between the responses in these two situations, demonstrating good construct validity.

Many studies and reviews suggest that there are no significant differences in the psychological functioning of patients with craniofacial anomalies as compared to general population norms. However, these studies report some difficulty in a particular area of functioning [25, 26].

There are substantial proofs of appearance concern due to poor facial appearance in this population and dissatisfaction with facial appearance [27, 28]. This dissatisfaction with facial appearance may lead to behavioral problems [29, 30]. However, others contradict this fact and suggest that subjects are pleased with their facial appearance [31].

Many studies point out that the adult population is at the risk of psychosocial problems due to facial appearance concerns [32] as compared to children who have shown good or even high levels of self-esteem, as reported by some authors [31]. The results of this study have supported the hypothesis that adult subjects with craniofacial anomalies have greater psychosocial impact due to facial and dental appearance. Facial and dental esthetics overlaps each other considerably as the former is influenced by the latter. In the current study, both DAS and PIDAQ scores were significantly higher in patients than controls, thus indicating the considerable psychosocial impact of facial and dental esthetics (Table 7).

Further, Versnel *et al.* stated that gender was not a determinant of satisfaction with the facial appearance in their study titled “Satisfaction with the facial appearance and its determinants in adults with severe congenital facial disfigurement” [10]. It was interesting that there was a statistically significant difference between the overall psychological impact of dental aesthetics and specific psychological impact in males and females, with females having higher scores (Table 8a). This may be explained by the fact that females are more concerned and dissatisfied with their dental appearance as compared to males. There was no effect of the residential area on the psychological impact of facial and dental appearance in patients. This was in accordance with Versnel *et al.* regarding facial appearance [10], and there is no literature report on dental appearance in this regard. However, controls showed significant differences in terms of the effect of the residential area with the negative self-concept (NSC) sub-domain of DAS59 and PD4 (aesthetic concern) sub-domain of PIDAQ with urban individuals having higher scores for DAS59. In comparison, rural subjects had higher scores for PD4, both of which indicate a poor psychosocial adjustment in subjects from rural areas. There was a significant relationship between age of presentation with DAS59, with all domains exhibiting a positive correlation, except NSC, which displayed a negative one. These findings are in contrast with the results reported by Thomas *et al.*, who have shown that adolescents are more dissatisfied with their facial appearance than young adults. These differences may be attributed to cultural, ethnic, and social differences between the populations studied. However, there was no significant relationship found regarding dental appearance. This was in accordance with Tin-Oo *et al.*, who found no relationship between age and satisfaction with dental appearance, stating that dental looks are important to younger and older adults [36]. This might be due to the great desire of looking attractive and young in males and females, especially in the current times, considering the role of media in promoting this aspect. In controls, age was related positively to negative self-evaluation regarding their facial appearance in contrast to patients, where it was negatively correlated, probably explained by the fact that controls (individuals with normal facial appearance) with facial changes due to aging evaluate their facial appearance negatively.

In the current study, there was no influence of the educational level on the psychological impact of facial appearance; however, it was related to dental aesthetics (the three sub-domains: PD2, PD4, PD5) and the total PIDAQ score. The relation was positive, but an increase in the educational level was negatively related to these sub-domains and total PIDAQ scores. This means that patients with higher education showed better psychological adaptation to dental appearance and had a lower score. This is in accordance with Versenal *et al.*, who stated in a recent study that subjects with a higher educational level are more satisfied with their appearance because they are more realistic and understand that a normal appearance might be difficult to achieve. However, as stated above, this was not true for facial appearance in this study and could be explained based on the different ethnic, cultural and social settings. In controls, the educational level is negatively related to SSC and positively related to NSC and was insignificant regarding the other parameters. This means that a higher educational level was associated with decreased social self-consciousness, which could be explained by the fact that individuals who attain higher education have more social interaction and have developed enough self-confidence compared to less-educated individuals.

However, a positive correlation with the negative self-concept was difficult to explain and it may suggest that despite their higher education and good social skills, these individuals evaluate themselves more negatively because they have a wider social circuit comprising normal people, making them feel different.

There were no significant differences in severity between the assessment of a specialist and that of the subject. This shows that there is an agreement between the clinicians and subjects regarding the severity of their condition. Subjects who rate their appearance problem as more severe have higher scores than those who rate it less severe. Further, the same is true regarding the objective assessment by clinicians. However, the psychological adaptation was more closely related to the subjective assessment. This is in accordance with Moss *et al.*, who demonstrated a linear relationship between subjective adjustment and severity, with greater perceived severity associated with poorer adjustment [13]. Similarly, he also demonstrated a weak but statistically significant quadratic relationship between objectively-rated severity and adjustment. The subjective severity was related to the psychological adaptation to dental appearance, specifically with the third sub-domain – the psychological impact. The expectations of patients seeking treatment are significant factors for the success of the treatment and satisfaction of the patient with the final outcome. This study shows that there are significant differences in the expectation of patients with craniofacial anomalies as compared with controls. 

Regarding the type of orthodontic treatment, patients with craniofacial anomalies are more unaware of the type of braces they may receive; expect that there are fewer chances that their teeth will be extracted, they are more likely to receive removable braces and fewer chances for any jaw surgery as compared to controls. These differences may be due to multiple surgeries for correction of their deformity, and they may favor removable braces as fixed braces could be further detrimental to their already compromised facial esthetics.

Regarding the duration of treatment, there was a significant difference, and patients with craniofacial anomalies estimated a long duration of orthodontic treatment as compared to controls. This may be because patients with craniofacial anomalies regularly visit the hospital from a very early age and could have gone through a long multispecialty treatment; therefore, they expect this treatment to be relatively long.

Patients believe that orthodontic treatment will improve their chances for a good career and will give them more confidence in society compared to controls. This may be due to the fact that they have a great desire to improve their overall facial and dental appearance, and they consider it as an important factor in their social interactions.

## Conclusion

This study found a significant psychological impact of altered facial and dental appearance in patients with craniofacial anomalies. Still, there was a non-significant effect of gender, locality, and educational level on the psychological impact of facial esthetics. However, regarding dental esthetics, patients with a low educational level showed a considerable psychological impact. Also, a significant psychological impact of dental appearance was seen in females. The significant effect of presentation age on the psychological impact of facial esthetics with an increase in age is associated with increased psychological impact, except for the negative self-consciousness parameter. However, it was not related to dental aesthetics. We found that only subjective severity was associated with the psychological impact of dental appearance.

## Acknowledgements

### Ethical approval

The approval for this study was obtained from the Ethics Committee of Patna Dental College (Approval ID: 410/4-2017).

### Consent to participate

Written consent was obtained from each participant.

### Conflict of interest

The authors declare that there is no conflict of interest.
